# Tinea gladiatorum due to *Trichophyton tonsurans* in a school wrestling team in Mexico: A case series

**DOI:** 10.18502/cmm.6.4.5439

**Published:** 2020-12

**Authors:** Alexandro Bonifaz, Javier Araiza, Andrés Tirado Sánchez, Adriana Barbosa Zamora, Alexander Gómez Sáenz, Andrea Méndez Juárez

**Affiliations:** 1 Dermatology Service, Department of Mycology, Hospital General de México “Dr. Eduardo Liceaga”, Mexico City, Mexico; 2 Department of Internal Medicine, Hospital General de México “Dr. Eduardo Liceaga”, Mexico City, Mexico

**Keywords:** Healthy carrier, Tinea corporis, *Tinea gladiatorum*, *Trichophyton tonsurans* Wrestlers

## Abstract

**Background and Purpose:**

Tinea gladiatorum is a type of dermatophytosis that occurs in combat athletes, such as wrestlers and judo fighters, as a result of Trichophyton species. Herein, we aimed to present a small outbreak of tinea gladiatorum in a high school in Mexico.

**Materials and Methods:**

Seven individuals belonging to the school fighting team were mycologically studied with direct examinations and cultures. In four cases, *T. tonsurans* was isolated and identified by morphological and proteomic methods (Matrix-assisted laser desorption/ionization- time-of-flight mass spectrometry). Out of the four subjects, two cases had clinical lesions presented as tinea corporis, and two cases were healthy carriers. *Trichophyton tonsurans* was also isolated from one of the four training mats (25%). All positive patients were treated with systemic or topical antifungals and achieved clinical and mycological cure.

**Conclusion:**

We report the first outbreak of tinea gladiatorum caused by *T. tonsurans* among a group of high school wrestlers in Mexico.

## Introduction

Tinea gladiatorum, also known as trichophytosis gladiatorum [ 1 ], is a common dermatophytosis in gladiators or fighters. *Trichophyton tonsurans*, an anthropophilic dermatophyte, is the most common etiologic agent of this infection [ 2 - 4 ]. The disease is transmitted by human-to-human contact; accordingly, it is common in contact sports, such as wrestling and judo (incidence of 24%) [ 1 ]. It is more prevalent among the individuals aged ≤ 20 years [ 2 , 3 ]. 

The clinical picture of this disease includes classical tinea corporis [ 4 , 5 ], tinea capitis [ 6 ], and exceptionally dermatophytic (Majocchi's) granuloma [ 7 ]. It presents with erythematous-squamous plaques with a raised and itchy border, mainly affecting the head, neck, and arms (65%), and rarely the lower limbs [ 1 , 2 , 8 ]. However, asymptomatic carriers have been also reported [ 5 ]. This disease is commonly observed worldwide [ 8 , 9 ]; however, in Mexico, the disease could be underreported since there are no previous reports of tinea gladiatorum. Herein, we present the first outbreak of this infection among high school wrestlers in Mexico. 

## Materials and Methods

**Figure 1:**
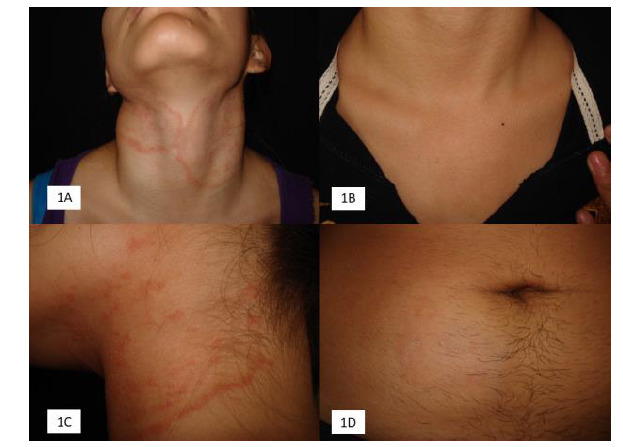
A & B) Extensive tinea gladiatorum (patient 1), B) post-treatment stage (Patient 1), C) close-up of the tinea gladiatorum (Patient 1), and D) hypochromic plaque with desquamation on the abdomen (Patient 5)

The case study began with a 20-year-old female university student who presented with a disseminated dermatophytosis, affecting the head at the right side of the face, neck, lateral and anterior face, and torso. The dermatosis consisted of 2- to 3-mm erythematous and fine-scaling papules with annular plaques having a raised border. The patient reported intense itching in the past 2 months. She practiced college wrestling. She was diagnosed with tinea corporis (Figure 1 A, B), which was confirmed by mycological studies and direct examinations of the scale with KOH (10%), revealing multiple thin and septate hyphae. For the purpose of examination, *T. tonsurans* was isolated in a Sabouraud dextrose agar (BD-DIFCOTM, Mexico) and then incubated at 28°C (Figure 1 C, D). The dermatophyte was identified by its morphological features and reproduction forms and then confirmed by matrix-assisted laser desorption/ionization/time-of-flight mass spectrometry (MALDI-TOF-MS; Vitek bioMèrieux®, Durham NC, USA). Treatment with oral terbinafine at a dose of 250 mg/day for 3 weeks resulted in a clinical and mycological cure. 

Based on anamnesis and patient's sport activity, it was decided to investigate the members of the school fighting group to which the patient belonged. In line with the research ethics considerations, the purpose and benefits of the study were explained to the students of the high school wrestling team, as well as their parents and teachers. Our institutional research ethics committee reviewed and approved the study protocol. Furthermore, the study was conducted in accordance with the Declaration of Helsinki Principles. 

In search of active lesions, some samples were collected from the various parts of the body in order to identify healthy carriers. For clinically suspicious lesions, the samples were subjected to fresh examinations (KOH) and cultured on Sabouraud dextrose agar with and without antibiotics. The samples were obtained from five parts of the body (i.e., scalp, neck, trunk, arms, and legs), using the sterile cytobrush technique. Additionally, some samples were taken from the four training mats using the same technique. The identification of the fungi was based on their macroscopic and microscopic characteristics, as well as MALDI-TOF MS [ 10 ]. In case of active lesions (with positive KOH), the patients were prescribed to use sertaconazole cream for 21 days. Furthermore, in the hairy areas and scalp, shampoo with ciclopirox/zinc pyrithione/keluamide was administered every 3 days for 30 days, without relapse. 

## Results and Discussion

**Figure 2:**
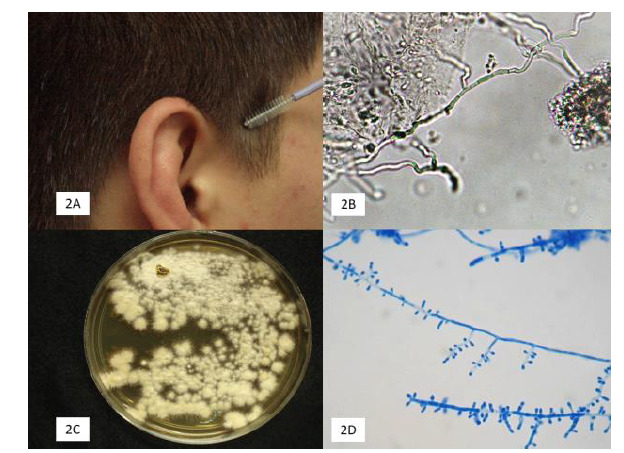
A) Taking a scalp sample with cytobrush technique in an asymptomatic carrier patient, B) hyphae on direct examination (KOH 10%, 40X), C) culture of *T. tonsurans* (Sabouraud dextrose agar, 15 days), D) micromorphology of *T. tonsurans* with a predominance of microaleurioconidia arranged in a “cross of Lorraine” (Cotton blue, X40)

Seven individuals (i.e., the female patient first referring and six male subjects from the school wrestling team) were included in the study. They had an age range of 17-21 years (mean=18.8 years). *Trichophyton tonsurans* was isolated from 4 (57.3%) subjects. Tinea corporis lesions were found in 2 (28.5%) cases, one of whom had extensive lesions, while the other had localized ones. However, no lesion was observed in the other 2 (28.5%) subjects, and dermatophyte was isolated from the inguinal region and scalp, without any clinical activities or symptoms. *Trichophyton rubrum* was also isolated from the interdigital skin of the toes in one patient. The three individuals with isolated dermatophytes finished the treatment without complications and achieved clinical and mycological cure. No *Trichophyton* species was isolated from the asymptomatic carrier. However, *T. tonsurans* isolate was found in one of the four training mats (25%) (Figure 2). 

Tinea gladiatorum is one of the most frequent conditions among contact athletes; accordingly, it is the cause of sports sanction for preventing outbreaks [ 11 ]. Skin-to-skin transmission is the most likely contagion mechanism associated with pre-existing skin lesions [ 1 , 8 ]. However, this disease can also be acquired through fomites (e.g., combs, clothing, and razors) [ 5 ]. In this regard, there are reports regarding the isolation of *T. tonsurans* from practice mattresses [ 2 , 4 ]. 

In Mexico, tinea corporis has the frequency of 15-25%, and its main etiological agents include T. rubrum (adults) and *T. tonsurans* (children), and to a lesser extent *T. mentagrophytes* [ 12 ]. This study is the first research in Mexico addressing tinea gladiatorum outbreak. In a recent study, Kermani et al. [ 13 ] conducted a systematic review and meta-analysis on 13 studies from Turkey, Iran, and the United States. In the first two countries, hand-to-hand fighting is a national sport. The study included 4,818 fighters, out of whom 391 cases were positive for* Trichophyton* species, with a prevalence of 2.4-90.62%. In our study, the small sample size did not allow population inferences. 

In the present study, out of the seven high-school wrestlers examined, *T. tonsurans* was isolated from 4 (57.14%) cases. Out of this group, two subjects had lesions matching tinea (28.5%), and the other two cases were asymptomatic carriers (28.5%), which is similar to previous reports [ 5 , 6 , 8 ]. We did not find published reports on tinea gladiatorum in Mexico because contact sports, including judo and hand-to-hand fighting, are not a common practice in Mexico. 

In the present research, *T. tonsurans* was isolated from all affected cases, which is consistent with the literature, reporting an association of up to 92% with tinea gladiatorum [ 2 , 4 , 11 ]. However, other species of* Trichophyton* genera have also been isolated, including *T. rubrum, T. mentagrophytes, T. verrucosum* [ 2 ], and *T. violaceum* [ 14 ]. Other dermatophytes, such as *Epidermophyton floccosum* and *Microsporum canis*, may rarely develop tinea gladiatorum [ 14 ]. 

We observed a predominance of tinea corporis, which is in line with the previous studies reporting this condition in 62.2% of cases [ 14 ]. In the present study, dermatophyte was isolated from the scalp of an asymptomatic carrier. This may increase the risk of reinfection [ 4 ]. 

In the current research, *T. tonsurans* was isolated from 1 (25%) out of 4 of the school mats, made of plastic. The examined mats were different from the traditional thick and rough fabric (esparto, palm, or reed) mats [ 11 ] that retain fungal spores. These mats are frequently used in countries, such as Turkey [ 6 , 9 , 15 ] and Iran [ 2 , 13 - 16 ]. In a previous study, skin-to-skin contact was proposed as the most likely mode of transmission since the isolation of the fungus from the mats was not possible; accordingly, they recommended periodic check-ups as necessary measures [ 16 ]. Benzalkonium chloride is useful for the effective disinfection of contaminated areas [ 17 ]. 

In the current study, the exact identification of species was accomplished using MALDI-TOF MS [ 10 , 18 ]; therefore, molecular diagnostic studies of the fungal colonies were not necessary. The antifungal susceptibility of *T. tonsurans* may vary. In this regard, in a recent report [ 19 ] conducted on 128 strains isolated from tinea gladiatorum based on an in vitro measurement method (CLSI broth microdilution document M38-A2), such topical antifungals as tolnaftate (0.022 µg/ml) and butenafine (0.088 µg/ml) had the lowest geometry mean minimum inhibitory concentration (MIC). Moreover, among systemic antifungals, itraconazole (0.026 µg/ml) and terbinafine (0.033 µg/ml) showed the lowest MIC, while fluconazole had the highest MIC value (12.540 µg/ml) [ 19 ]. 

Tinea gladiatorum is commonly treated with systemic antifungals, mainly terbinafine, itraconazole, or fluconazole, within short periods of 15-30 days [ 11 ]. Adams et al. [ 8 ] reported that a weekly dose of 200 mg of fluconazole for 4 weeks may effectively treat infection and allow negative cultures in all infected wrestlers. However, it is important to mention that this antifungal has the highest MIC, and the use of itraconazole and terbinafine may give better results in clinical settings [ 19 ]. Other regimens reported with this triazole are 100 mg/week or 100 mg/day for 3 days [ 20 ]. 

Concerning our experience, the initial patient was managed with oral terbinafine, one of the most effective antifungal agents against* Trichophyton* species [ 4 , 6 , 7 ]. In contrast, the other two cases were managed with sertaconazole cream because they had localized lesions, while the asymptomatic carrier with the affected scalp was managed with a ciclopirox and zinc pyrithione shampoo. 

## Conclusion

Tinea gladiatorum is exceptional in Mexico, and this study represents the first report in this country. We reported a small outbreak in students who practiced hand-to-hand combat. In the present study, *T. tonsurans* was isolated from all cases. 

## Author’s contribution

A. B. Designed and reviewed the study. J. A. and A. G. S. Performed mycological identification. T. S. Wrote, edited, and revised the study. A. B. Z. and A. M. J. Performed clinical and therapeutic study and reviewed the paper.

## Financial disclosure

There are no financial conflicts of interest to disclose.
